# Lorraine Strain of *Legionella* *pneumophila* Serogroup 1, France

**DOI:** 10.3201/eid1404.070961

**Published:** 2008-04

**Authors:** Christophe Ginevra, Françoise Forey, Christine Campèse, Monique Reyrolle, Didier Che, Jerome Etienne, Sophie Jarraud

**Affiliations:** * Université de Lyon, Lyon, France; † Institut National de la Santé et de la Recherche Médicale U851, Lyon, France; ‡ Hospices Civils de Lyon, Bron, France; § Institut de Veille Sanitaire, Saint-Maurice, France

**Keywords:** *Legionella*, epidemiology, endemic strain, emergence, letter

**To the Editor:** Legionellosis is a pneumonia caused by inhalation of *Legionella* spp. in aerosol water particles. *Legionella pneumophila* is responsible for ≈90% of cases; serogroup 1 alone accounts for ≈85% of cases (*1*). Epidemiologic analyses based on pulsed-field gel electrophoresis (PFGE) and sequence-based typing of clinical isolates of *L. pneumophila* serogroup 1 have detected sporadic, epidemic, and endemic strains (*2*). Most cases are sporadic and are associated with strains that have not been identified. A strain is considered endemic to an area when several isolates that have identical PFGE patterns and that cause several epidemiologically unrelated cases of legionellosis are detected in that area. Since 1998, the most prevalent strain endemic to France has been the Paris strain (*3*), which was responsible for 12.2% of culture-confirmed cases of legionellosis from 1998 through 2002 (*3*). The Paris strain has also been detected in clinical samples from several other European countries (Switzerland, Italy, Spain, and Sweden) and in environmental samples (*3*,*4*).

We identified a new endemic clone of *L.* *pneumophila* serogroup 1, the Lorraine strain, and report its spread throughout France. The French national reference center for *Legionella* collects all clinical isolates of *Legionella* spp. as part of an epidemiologic surveillance system. All *L. pneumophila* serogroup 1 isolates are typed by PFGE methods as described (*4*). When necessary, sequence-based typing (*5*,*6*) and monoclonal antibody–based (MAb) subgrouping are also used (*7*).

From 1995 through 2006, the reference center typed 1,768 clinical *Legionella* isolates by means of PFGE. Most PFGE patterns were unique and thus corresponded to sporadic cases. Another 145 (8.2%) patterns were identical and corresponded to the endemic Paris strain. An identical PGFE pattern was also found for 80 (4.5%) isolates from epidemiologically unrelated patients; these isolates were further characterized by sequence-based typing and MAb subgrouping. Sequence type was deduced for the following genes: *flaA, pilE, asd, mip, mompS, proA,* and *neuA* (*6*). The sequence type was obtained for 78 of the 80 isolates and was 5, 10, 22, 15, 6, 2, 6. The sequence type of the remaining 2 isolates differed from that of the other 78 by 2 alleles (*pilE* and *proA*) and was 5, **1**, 22, 15, 6, **10**, 6 (**boldface** indicates differences). All but another 2 isolates (which belonged to the Benidorm subgroup) belonged to the Allentown MAb subgroup. Hence, the new endemic strain, Lorraine, was represented by 76 isolates that had an identical PFGE pattern, sequence type, and MAb subgroup.

Isolation of the Lorraine strain was reported anecdotally before 2002. Since 2002, the prevalence of this strain in France has increased considerably, accounting for 10.5% clinical isolates in 2005 and 9.0% in 2006 (Figure). In contrast, prevalence of the Paris strain was ≈10% from 1998 through 2002 and peaked in 2000 (16.9%) in association with a hospital outbreak in Paris. From 2003 through 2006, prevalence of the Paris strain fell to ≈6.5%.

**Figure:**
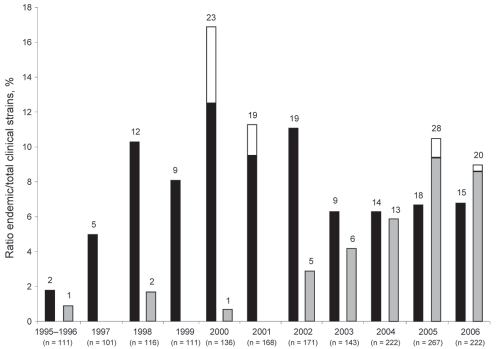
Prevalence of the *Legionella pneumophila* Paris (black bars) and Lorraine (grey bars) endemic strains, France, 1995–2006. White bar sections represent the proportion of strains isolated during outbreaks. For example, in 2000 the Paris strain accounted for 16.9% of clinical isolates: 12.5% unrelated and 4.4% related to the same outbreak. Numbers above each bar indicate the number of isolates.

The Lorraine strain has caused 2 outbreaks. In the first, 3 isolates were recovered from respiratory samples of 34 patients for whom legionellosis was diagnosed by urinary antigen testing in Lyon in 2005. The second outbreak occurred in a western suburb of Paris in 2006, when 1 isolate was cultured from respiratory samples of 12 patients whose diagnoses were also made by urinary antigen testing.

From 1995 through 2006, >4,000 environmental *Legionella* isolates in France were typed by PFGE, and >700 types were identified. The Paris strain type was identified >500 times, but the Lorraine type was identified in only 3 water samples, including 1 from the cooling tower responsible for the outbreak in the Paris suburb. The Lorraine strain is thus rarely found in water samples, which hinders environmental investigations of its sources in outbreaks of legionellosis.

A similar disparity between the clinical and environmental distribution of *Legionella* strains has been reported (*8*). In a collection of 284 unrelated clinical isolates and 117 unrelated environmental isolates, Harrison et al. found that 3 types, identified by restriction fragment length polymorphism, accounted for 40% of clinical isolates but only 18% of environmental isolates (*8*).

The high prevalence of the Lorraine strain in clinical samples and its extremely rare detection in water samples have several possible explanations: 1) this strain could be related to specific host factors; 2) it could be highly virulent even in low amounts, below the culture detection limit; and 3) it could be more susceptible than other strains to different stressors (e.g., biocide treatment, selective preplating techniques, environmental medium specific components).

In conclusion, prevalence of a new *L. pneumophila* serogroup 1 strain, Lorraine, endemic to France, is increasing in clinical samples although rarely detected in water samples. The type strain, Lorraine (CIP108 729), is available from the strain collection of the Pasteur Institute (Paris, France).

## References

[1] Doleans A, Aurell H, Reyrolle M, Lina G, Freney J, Vandenesch F, Clinical and environmental distributions of *Legionella* strains in France are different. J Clin Microbiol. 2004;42:458–60. 10.1128/JCM.42.1.458-460.2004 14715805 PMC321724

[2] Aurell H, Farge P, Meugnier H, Gouy M, Forey F, Lina G, Clinical and environmental isolates of *Legionella pneumophila* serogroup 1 cannot be distinguished by sequence analysis of two surface protein genes and three housekeeping genes. Appl Environ Microbiol. 2005;71:282–9. 10.1128/AEM.71.1.282-289.2005 15640199 PMC544207

[3] Aurell H, Etienne J, Forey F, Reyrolle M, Girardo P, Farge P, *Legionella pneumophila* serogroup 1 strain Paris: endemic distribution throughout France. J Clin Microbiol. 2003;41:3320–2. 10.1128/JCM.41.7.3320-3322.2003 12843082 PMC165293

[4] Lawrence C, Reyrolle M, Dubrou S, Forey F, Decludt B, Goulvestre C, Single clonal origin of a high proportion of *Legionella pneumophila* serogroup 1 isolates from patients and the environment in the area of Paris, France, over a 10-year period. J Clin Microbiol. 1999;37:2652–5. 10405416 10.1128/jcm.37.8.2652-2655.1999 PMC85305

[5] Gaia V, Fry NK, Afshar B, Luck PC, Meugnier H, Etienne J, Consensus sequence-based scheme for epidemiological typing of clinical and environmental isolates of *Legionella pneumophila.* J Clin Microbiol. 2005;43:2047–52. 10.1128/JCM.43.5.2047-2052.2005 15872220 PMC1153775

[6] Ratzow S, Gaia V, Helbig JH, Fry NK, Luck PC. Addition of *neuA*, the gene encoding N-acylneuraminate cytidylyl transferase, increases the discriminatory ability of the consensus sequence-based scheme for typing *Legionella pneumophila* serogroup 1 strains. J Clin Microbiol. 2007;45:1965–8. 10.1128/JCM.00261-07 17409215 PMC1933043

[7] Helbig JH, Bernander S, Castellani Pastoris M, Etienne J, Gaia V, Lauwers S, Pan-European study on culture-proven Legionnaires’ disease: distribution of *Legionella pneumophila* serogroups and monoclonal subgroups. Eur J Clin Microbiol Infect Dis. 2002;21:710–6. 10.1007/s10096-002-0820-3 12415469

[8] Harrison TG, Doshi N, Fry NK, Joseph CA. Comparison of clinical and environmental isolates of *Legionella pneumophila* obtained in the UK over 19 years. Clin Microbiol Infect. 2007;13:78–85. 10.1111/j.1469-0691.2006.01558.x 17184291

